# The Philanthropist's Vade Mecum

**Published:** 1888-12-29

**Authors:** 


### THE PHILANTHROPIST'S YADE MECUM.

TWENTY-FIVE years of observation and experiment have convinced us of the error into which many managers of charities fall at this season. They prepare most telling appeals, involving much thought and ingenuity, and at great expense. They then select the classes to whom they will address themselves, and about ten days or a week before Christmas the bombardment of the charitable is commenced from every quarter of the compass, and by every class of charity. Much of the force of these appeals is lost from the circumstance that the recipients are often so overwhelmed by the number which each day's post delivers on their tables that, like the prospectuses of company promoters, coal circulars, and similar productions, they are cast unread into the fire or the wastepaper basket, whichever happens to be handiest. Knowing all this, we have not issued any Christmas appeal number, but now that Christmas is over, and everybody has time to look round and act, we have pleasure in presenting to our readers and the public the many weighty reasons why they should give some of their abundance to the medical charities which sadly need their help. Those philanthropists who agree with the views here expressed, and who wish to be relieved from some of the worries of Christmas appeal letters for the future, might request the secretaries of each institution they contribute to, to acknowledge the receipt of the money in the columns of THE HOSPITAL. This would be a delicate hint which the able appealist would not fail to follow in years to come, and we commend it, therefore, to the consideration of philanthropists as a class.

It will be observed that our advertising columns contain very few appeal notices, the reason being that THE HOSPITAL exists to aid, not to live upon charities. Those who recognise that our columns are read by some forty thousand persons every week, the majority of whom, at any rate, have a deep sympathy with every good philanthropic agency, have not been slow to adopt the view of the late lamented Major Ross, M.P., the able Chairman of the Middlesex Hospital, who maintained "that one small advertisement, costing very little, inserted regularly in a paper devoted to the interests which the particular charity tended to promote, was worth all the special advertisements costing heaps of money, which are inserted by some in any periodical which issues a special Christmas number. Spread the money so spent over the entire year and you will reap abundantly instead of spasmodically." We believe in the wisdom of this contention also, and hence our publisher has not given any special prominence to the fact that this issue, which philanthropists cannot fail to widely read and carefully preserve, has not been announced long in advance, but is submitted on its merits for the candid consideration of those whom it may concern. We are glad to receive the advertisements of any reputable charity, but we have been obliged, on public grounds, to refuse insertion to some which have been offered to us.

It will be observed that we give the name of the Matron as well as the Secretary in every instance where the information is available. This has been done because it is recognised that the more people can be induced to visit the hospitals, and thus to gain a personal insight into the work which goes on there, the better for the people, the institutions, and the public at large. It is not only men who are wanted in much greater numbers to visit the hospitals and take part in their management, but women also, who are never more happily employed than in ministering to the sick and the sorrowful. We have thought that ladies would like to know the Matrons, and to have an opportunity of seeing the work under their guidance, rather than that of the Secretary, who will have his hands full, we hope, in attending to the sterner sex. In any case, the information will prove of permanent value for reference, and it will be seen that an endeavour has been made to give a new fact about most institutions dealt with, so that the whole of what follows may form interesting and instructive reading.

Finally, it has of course been impossible to deal with the provincial institutions for want of space. We have, however, dealt with the claims of many of them week by week, as the reports and information have been sent to us, and we shall continue to do this in the future. It may further be pointed out that *The Hospital Annual*, which will be issued next month, will contain full particulars, of all classes of medical charities and nursing institutions in the country, forming, as it will do, a year-book of reference on the subject, and so prove of permanent interest and value. It will also deal with convalescent institutions, the managers of which do not seem to issue Christmas appeals, a wise forbearance on their part which ought to be rewarded in the Spring, when they have to make an appeal to enable them to efficiently discharge the useful work which devolves upon them. We have only to add that we will gladly acknowledge any contributions which may be sent to the various hospitals and other charities, for which it is our pleasant duty and privilege on this occasion to appeal. We believe that many philanthropists will be grateful that we have delayed issuing this Special Number until after Christmas, when the more social and personal duties have happily been fulfilled, and everyone has leisure, and we hope inclination, to attend to outside claims in a liberal and large-hearted spirit. At any rate, we urge all to read the following brief but critical review of our great charities, of which all may well be proud. "'Twere good you do so much for charity."

### GENERAL HOSPITALS.

**Charing Cross Hospital.**—This institution is situated in the centre of the most crowded thoroughfares in the metroplis, and has therefore to provide for a greater number of accident, *i. e.* urgent cases, than probably any other hospital of its size. All such cases are immediately admitted without delay or difficulty. Can any stronger appeal be made than this to the many visitors who usually come to London, or to the public generally? We think not. Yet, the need for pecuniary help is great and pressing, for although the expenditure last year exceeded the available income by £3,000, fifty beds were unoccupied for want of funds. Secretary, Mr. Arthur E. Reade; Matron, Miss Gordon.

**Deaconesses' Institution and Training Hospital**, Tottenham.—The Princess of Wales lately opened a new wing of the hospital presided over by these sisters; and the work is growing in every direction and sorely needs support. There are at present 103 beds, and the in-patients numbered 721 last year; each patient costing 2s. 10d. per day, and staying on an average 31½ days. The sisters do the dispensing, and attend to the domestic departments; one acts as secretary. The medical staff give gratuitous attention, so that the strictest economy is maintained everywhere, and all donations benefit the patients solely.

**French Hospital,** 10, Leicester Place, W.C.—Open to all foreigners, it commends itself especially to the large French-speaking population of London, to whose liberality we earnestly commend it. Hon. Secretaries, Messrs. Ernest Ruffer and F. Lovel.

**German Hospital,** Dalston, E.—This hospital is one of the most economically-managed of all, a fact which gives no little satisfaction to its president, H.R.H. the Duke of Cambridge. It does its work quietly, with exceptional efficiency; all accident cases, of whatever nationality, are admitted free, and it deserves much more general support than it has hitherto received. Secretary, Mr. C. Feldmann; Matron, Miss Christiane Burger.

**Great Northern Central Hospital.**—This is a new hospital, which is to contain 150 beds; quite free, utterly unendowed, and with a debt to discharge. During November 3,322 cases were attended to, of which 209 were accidents. Nine in and 80 out patients were not able to be admitted for want of room. To carry on the great and beneficial work of this hospital, donations and subscriptions are earnestly asked. Secretary, Mr. William T. Grant.

**Guy's Hospital.**—We have heard a great deal about Guy's Hospital lately, and its claim for aid has been recognised by the Hospital Sunday Fund, which has for the first time given it a grant. Guy's is too old a friend of the public to need much said in its favour, only so long as it has closed wards and is crippled by poverty it is a reproach to the metropolis. Help has been forthcoming lately, and things look better; a little extra exertion this Christmas, and Guy's might once more throw wide open every ward. Superintendent, Dr. Steele; Matron, Miss Jones.

**King's College Hospital.**—This institution is in urgent need, for whereas the total annual expenditure in 1887 was upwards of £25,000, the total income was only rather more than £13,000, *i.e.* very little more than half what was required to enable it to do its work with efficiency. It had fifty vacant beds throughout the year, a sad evidence of public forgetfulness, remembering what its famous medical school has done for all classes of the community. Secretary, Mr. J. Mosse Macdonald; Sister-Matron, Miss Monk (Sister Katherine).

**London Hospital.**—This is the greatest hospital in this country. It includes special departments under eminent medical men for the treatment of all classes of disease, the number of beds devoted to children being greater than those to be met with in most children's hospitals. The character of the work done and its value to the public may be realised from the statement that the able matron, Miss Luckes, has three assistants under her, in addition to two night superintendents, 19 day sisters, and 220 staff and probationer nurses. This great hospital of the East End is in serious want of funds, as the committee depend on voluntary contributions for £34,000 a-year, to enable them to maintain the 640 beds which are daily occupied by urgent cases. Secretary, Mr. G. Q. Roberts; Matron, Miss Eva C. Lückes.

**Miller Hospital,** Greenwich.—This was the first hospital built by the inhabitants of an outlying metropolitan district. There is urgent need that the bed accommodation should be at least doubled, and those who believe in decentralisation cannot do better than send a cheque without delay to the Hon. Secretary, Colonel Bristow.

**Middlesex Hospital** contains 307 beds, of which 34 are specially set aside for cancer cases. The assured income is only half the expenditure, which amounts to about £30,000 a-year. The weekly board of governors solicit the succour of the public to support this old-established hospital. Secretary, Mr. F, Clare Melhado Matron, Miss Thorold.

**Metropolitan Hospital,** Kingsland Road, E—This is an institution which should commend itself to the sympathies of all who believe in the provident principle as applied to medical relief. To Sir Edmund Hay Currie belongs the proud position of having successfully worked a general hospital in London upon the provident system. No more eloquent appeal could surely be made to the thoughtful philanthropist, who learns that pecuniary help and encouragement are here needed. Secretary, Mr. Charles H. Byers.

**North-West London Hospital,** 18, Kentish Town Road.—This is a small but vigorous institution, with 50 beds, which deserves encouragement. It is conducted on Mr. Spurgeon's principle, that the managers of a charity should never be waiters upon Providence, but that funds should be collected before work is lavishly undertaken. All thrifty people will, therefore, necessarily support this institution, which requires £1,000 a-year more in annual subscriptions to enable the managers to keep all the beds occupied. Secretary, Mr. Alfred Craske.

**Poplar Hospital for Accidents,** Blackwall, E.—This institution is entirely free, and we would strongly urge philanthropists to pay it a visit. If they do, we believe funds will be abundant during the ensuing year. Secretary, Lieutenant-Colonel Edward Feneran; Matron, Miss E. Pilcher.

**Royal Free Hospital,** Gray's Inn Road.—During the past year this hospital has increased the accommodation for the nursing staff, which has entailed a considerable enlargement of the yearly expenses; parts of the building are now so old that it is absolutely necessary for the work of rebuilding to be undertaken without delay, and if the public respond to the appeal now issued, operations will be commenced this year. The total expenditure of the hospital reaches £11,000 a-year, the assured income is £1,500, so that £10,000 has to be raised by constant appeals to the benevolent public. Nearly two million patients have been benefited since the Royal Free was founded, and last year the in-patients numbered 1,836, and the out-patients 23,541. Secretary, Mr. Conrad W. Thies; Matron, Miss Barton.

**St. George's Hospital,** Hyde Park Corner.—Occupying a commanding position, there is reason to believe that it suffers in consequence, because most people feel that a hospital placed at Hyde Park Corner must necessarily be in funds. This is not the case, however, as the managers had to spend some £7,000 more than their total income to defray the cost of maintaining the 277 beds which were daily occupied last year. We hope that all who pass St. George's Hospital in future will contribute something to its funds in the course of the year. They could not do better. Secretary, Mr. C. L. Todd; Matron, Mrs. Coster.

**St. Mary's Hospital.**—The last year has seen a large addition to this popular hospital, in the shape of a new wing, erected by the late Mr. Stanford. This will greatly add to the annual expenses of the institution, so it is to be hoped that many new friends will come forward with donations and subscriptions this year. The hospital is not endowed, and has an annual expenditure of £20,000. In-patients 2,352, out patients 22,443. The total number of beds available is 281, and of these over 200 are occupied on an average. The increased accommodation will be a great boon to the patients if only funds are forthcoming to keep it in working order. Secretary, Mr. Thomas Ryan; Matron, Miss Medill.

**Seamen's Hospital (Dreadnought) Society,** Greenwich.—After Mr. Michelli's vigorous abuse of hospital extravagance, it can be taken for granted that this society, of which he is secretary, is managed with the utmost economy. The hospital is open day and night to sailors of all nations, and is perfectly free. Seven thousand pounds have to be raised annually by appeals to the public, and it is hoped that this unique charity may not be forgotten at this season. The

Society maintains two free dispensaries for seamen—one at Gravesend and one at the London Docks. Secretary, Mr. P. Michelli; Matron, Miss Cooke.

**University College Hospital.**—This hospital pleads for funds both now and at all times, as it has an annual difference between its receipt and expenditure of £16,000 to make up every year. Luckily, the work of University College Hospital is well known, but more annual subscriptions would be very welcome. In-patients, 2,836; out-patients, 37,377. The Samaritan Fund relieves destitute patients, supplies surgical appliances, and sends convalescent patients to the seaside or the country. Secretary, Mr. Newton H. Nixon; All Saints' Sisters in charge of nursing.

**Westminster.**—This occupies the proud position of being the first of metropolitan hospitals having a medical school attached in point of economy, the average cost of each bed occupied being only £71 13s. 1d., including all the expenditure on out-patients, and under every other head. It is conveniently situated for visitors, and does an immense service to the country by training an exceptionally large number of excellent nurses. We hope its cash-book during the next few weeks will bear eloquent testimony to the genuine pride which the inhabitants of Westminster ought to take and do in their hospital. Secretary, Mr. Sidney N. Quennell; Matron, Miss Pyne.

**West London Hospital,** Hammersmith Road, W. —This institution has enforced Mr. Spurgeon's principle already referred to by closing its wards for certain months in each year, when the income proved insufficient to maintain the beds occupied. This step, however necessary, has caused criticism in certain quarters, and should therefore stimulate those who believe in the strict enforcement of what they hold to be sound principles of conduct. It contains 101 beds, and treats about 18,000 in and out-patients each year. Secretary, Mr. R. J. Gilbert; Lady Superintendent, Miss Hardy.

### HOSPITALS FOR CONSUMPTION.

**Brompton Hospital for Consumption.**—The pressure for admission to this hospital has had to be met by a new building, containing 137 additional beds, which represents an annual increase in the expenses of over £7,000. The assured income is under £3,000. This hospital is well known as one where the patients are well cared for, and every effort is made to brighten their clouded lives. There is a Samaritan Fund connected with hospital, which is often able to greatly benefit a patient by a few weeks by the sea. Fresh subscriptions are specially needed to maintain the new building. Secretary, Mr. H. Dobbin; Matron, Miss F. Abbott.

**City of London Hospital for Diseases of Chest,** Victoria Park, E.—Although this institution spent last year nearly £3,000 more than its total income, we find it impossible to state its present requirements, as it does not appear to advertise nor to send information of its work to the press. Such seclusion is not, however, calculated to bring financial strength to a voluntary charity, and we shall hope to receive communications for publication from the authorities during the year. If the public could be made to realise that the late Dr. Peacock did much of his invaluable work as physician to this hospital, funds would certainly flow into its exchequer. Secretary, Mr. T. Storrar-Smith; matron, Miss H. G. Hetherington.

**North London Consumption.**—This institution has been thoroughly reorganised and remodelled; and, thanks to the devotion of Mr. B. A. Lyon, the chairman, it has become one of the most useful of its class. Mr. Lyon is energetically supported by some of the most influential residents at Hampstead, where it is situated, and this growth of local opinion has proved invaluable to its interests in many ways. Funds are particularly needed to pay off a mortgage of £5,000, and to provide 12 additional beds; 3,900 out-patients and 350 in-patients treated yearly. The charity is unendowed, and in debt, and is hampered in its good work for want of increased annual subscriptions. No disease is a greater scourge amongst the poor than consumption; and to offer an asylum to those thus afflicted is a truly great and good work. Secretary, Mr. Lionel Hill, M.A.; Matron, Miss Blaxland.

**Royal Hospital for Diseases of the Chest,** City Road.—The buildings having been recently re-constructed, and the accommodation increased, the useful work done by this institution is more important to the public than ever. It contains 80 beds, relieves some 6,000 patients annually, and needs £1,700 a-year more in annual subscriptions, which will surely be forthcoming when it is stated that Mr. S. Hope-Morley is the chairman of the council; Secretary, Mr. John J. Austin; Matron, Miss M. L. Smith.

**Royal National for Consumption,** Ventnor.—This institution provides for a well-recognised want by maintaining some 150 beds on the *separate* principle at Ventnor, which are mainly occupied by metropolitan cases. All who have had cases of consumption in their families or amongst their friends must recognise the importance to the poor of the maintenance and extension of this institution. No cases are more distressing and none surely will demand and receive more substantial sympathy. Secretary, Mr. G. Morgan, 34, Craven Street, W.C.

### HOSPITALS FOR CHILDREN.

**East London Hospital for Children,** Shadwell, E.—An excellent institution, dear to the readers of Charles Dickens, whose memorable account of the old building will live for generations. Unfortunately, the children admitted are in as dire straits now as then, and funds are sadly needed. Secretary, Mr. Ashton Warner; Matron, Mrs. Fisher.

**Evelina,** Southwark Bridge Road, S.E.—There was great joy amongst the children in this hospital during Christmas week, owing to the announcement of a children's fête on the 28th inst., and to the fact that it was whispered about that the trees would be lighted soon after three o'clock. It is the only hospital in South London exclusively for children. Any poor sick or suffering child is admitted if there is room. It is open to visitors every day from two to four. Contributions, which could not be better bestowed, may be sent to Dr. George Carpenter, or to the Secretary, Mr. T. S. Chapman. Matron, Miss Alice Cross.

**Hospital for Sick Children,** Great Ormond Street, W.C.—This institution has prospered by leaps and bounds of late, owing to the energy and devotion of the lady superin tendent, Miss K. Philippa Hicks, and the secretary, Mr. Adrian Hope. It requires a good round sum for building purposes, and some thousands a-year additional income, and both should be promptly subscribed. To plead for children at Christmas should always be an easy task; and we hope our readers will remember that the new wing of this hospital is stopped for want of funds, and that sickly little ones are therefore denied the shelter they need. This charity is not endowed, but dep-nds entirely upon voluntary support. Visitors are welcome any afternoon if they will only go and see, and leave of their riches a trifle to aid the work there carried on.

**North-Eastern Hospital for Children.**—This institution has prospered greatly since Mr. Alfred Nixon became secretary some years ago. Over 1,000 children have been treated as out-patients at this hospital in Hackney-road, E., every week during the past year. The 55 beds in the wards are always full. Patients are admitted from all parts of London and the United Kingdom. The hospital is entirely dependent for support on voluntary contributions. Donations and new annual subscriptions are urgently required to meet the Christmas accounts, which are very heavy. Matron, Miss E. W. Curno.

**Paddington Green Children's Hospital**, W.—The growth of this institution in popularity and extent has been so rapid as to be remarkable even in these times of progress. It does an immense amount of good, and has recently opened a branch in the country for convalescent children needing fresh air, but requiring skilled nursing and surgical dressings, a feature in its work, which entitles it to special commendation and support. Secretary, Mr. H. W. Pearce; matron, Miss Anderson, who, we rejoice to hear, has made a good recovery, and is so able to resume her duties at Paddington Green.

**Victoria Hospital for Children**, Queen's Road, Chelsea.—Established at Chelsea in 1866 for the relief of the sick and suffering children of the poor, this unendowed hospital seeks further subscriptions to help it to carry on its work. There are 84 beds—70 at the hospital, and 14 at the convalescent branch at Margate. The number of in-patients last year was 599, out-patients 10,800. The expenditure, which is only 19s. per patient, yet reaches over £6,000 a-year, which has to be raised entirely by voluntary subscriptions. Now at Christmas, when so many children are enjoying healthy romps and holidays, a thought might be cast on those poor children whose fate is far otherwise, and whose sufferings might well call forth sympathy and generosity. Secretary, Captain W. C. Blount, R.N.; Matron, Miss Cooper.

### SMALLER, BUT NO LESS WORTHY INSTITUTIONS.

**Alexandra Hospital for Children**, 18, Queen Square, W.C., takes in cases of hip disease for prolonged treatment, and should command liberal supplies on this account alone. Secretary, Major J. L. Steavenson; Matron, Miss L. E. Moore; Lady Superintendent, Miss E. Jackson.

**Belgravia Hospital for Children**, 79, Gloucester Street, Pimlico.—Wealthy Belgravia ought to take this institution under its special care. Lady Superintendent, Miss Munro.

**Cheyne Hospital**, 46, Cheyne Walk, S.W.—This is for incurable children, those little patient sufferers, whom all the world must welcome an opportunity of helping, if only by a subscription. Hon. Secretary, Mrs. Wickham Flower.

**Home for Incurable Children**, 2, Maida Vale, W. — Excellently managed and greatly wanted, but sadly in need of funds. Acting Secretary, Miss Coleman.

### HOSPITALS FOR WOMEN.

**Chelsea Hospital for Women**, Fulham Road.—This hospital has 63 beds, and treats respectable poor women and ladies in reduced circumstances who are victims of ills peculiar to their sex. Contributing in-patients occupy separate wards, paying from 10s. 6d to 42s. a-week according to their means. Last year there were treated 457 in-patients, and over 3,000 out-patients. The hospital is entirely without endowment or reserve funds, and earnestly desires more annual subscriptions from women who have pity on their sex. Treasurer, Mr. Henry S. Wright.

**Establishment for Gentlewomen during Temporary Illness**, 90, Harley Street, W.—The idea of this institution is excellent, and its medical management should leave nothing to be desired, seeing that one of the kindest and best of surgeons, Mr. George Lawson, has for many years associated himself with it. It takes small payments from the patients, and for this reason is always in want of substantial help from wealthy people. Surely the ladies of England will not allow it to languish for want of funds. Lady Superintendent, Miss Meyrick.

**Grosvenor Hospital for Women and Children**, Vincent Square, Westminster.—This institution was founded on the principle that patients should contribute to their maintenance according to their means. The income thus derived amounts to one-third of the expenditure. It is a new (1886) and small hospital, which is growing in public favour. Superintendent, Miss K. Hughes.

**Hospital for Women**, Soho Square, W.—This institution has an excellent department for the admission of paying patients, from the payment of whom it derives a fourth of its total income. It meets a distinct want, and it would be well if the number of its beds (sixty-six) could be doubled, and that at least two-thirds of them should be made available for paying patients. There was a deficiency of nearly £1,500 last year, so that cheques will be welcomed, if sent, as we think they may be, in the conviction that the money will be wisely spent. Secretary, Mr. David Cannon; Matron, Miss Sutcliffe.

**New Hospital for Women**, 322, Marylebone Road, W.—Here all the physicians are women, and we are as surprised as we are sorry to find that the total available income is not adequate to keep the whole of the beds constantly occupied throughout the year. This institution ought to appeal especially to the gentler sex, for all the inmates, both patients and doctors, are women. Twenty-six beds are made up, and during the past year 263 in-patients and 5,163 out-patients were treated. The hospital is not endowed, and funds are earnestly asked towards securing more suitable premises. Secretary and Superintendent, Miss Margaret M. Bagster.

**Royal Hospital for Children and Women**, Waterloo Bridge Road.—Ably administered on kindly principles, in the best interest of the patients, and to the great comfort of their friends, who are chiefly the poorest of the poor. We have known of a case of a child which was regarded as well nigh hopeless, which was admitted to this hospital during the year, and which owes its life and restored health, under Providence, to the devotion displayed by the medical staff and the nurses. Situated in a poor neighbourhood, it appeals strongly to the sympathy of the really charitable, who could not do better than raise the annual subscriptions by an additional £500 per annum. Secretary and Superintendent, Mr. R. G. Kesting; Matron, Miss Nicholson.

**Samaritan Free Hospital**, 13, Lower Seymour Street, W.—Founded in 1847 for the relief of women and children, this charity is unendowed, and appeals for Christmas gifts to close the year free of debt. In-patients, 538; out-patients, 4,648. Secretary, Mr. George Scudamore; Matrons, Miss Butler and Miss Tidy.

### LYING-IN HOSPITALS.

**British Lying-in**, Endell Street, W.C.—This is a purely ticket hospital, all patients having to apply to subscribers for recommendations. It should therefore receive the support of the upholders of the old system of administering voluntary hospitals. Secretary, Mr. Fitz Roy Gardner; Matron, Miss E. Freeman.

**City of London**, City Road, E.C.—One of the best of its class. At this hospital, situated in a poor crowded district, 1,650 women were confined last year. The average expenditure is £2,585, and though the hospital is endowed, the expenditure is greater than the income. Secretary, Mr. R. A. Owthwaite, who has done good service for the hospitals of London in many ways; Matron, Miss S. E. Allen.

**General Lying-in,** York Road, S.E.—Recently re-organised, and has a training-school for midwives and nurses attached, keeps its expenditure within reasonable control and so deserves support. Matron, Miss Atkinson; Secretary, Miss Annie White.

**Mothers' Lying-in-Home,** Juniper Street, Shadwell, E.—As its name implies, this unpretending but most useful charity has done an infinity of good. We should be glad to hear that some of our readers, at any rate, had contributed liberally to its funds. Secretary and Lady Superintendent, Mrs. Ashton Warner, to whose energy and kindly thoughtfulness its existence is due.

**Queen Charlotte's Lying-in-Hospital.**—This is a school of midwifery and monthly nursing. There is an assured income of £415 to meet an expenditure of £5,165, so that subscriptions and donations are eagerly sought. The in-patients number 885, and the out-patients about one thousand in a year. The hospital is of double benefit to the poor, in that it receives suitable cases within its wards and trains nurses to wait on those who cannot gain admittance. Secretary, Mr. G. Owen Ryan; Matron, Mrs. Phillips.

**Royal Maternity Charity,** 31, Finsbury Square.—Under the patronage of Her Majesty the Queen, with Sir John Lubbock as treasurer, this charity stands secure above all doubt, and cannot be much abused. Last year four thousand poor women were the recipients of its bounty, and the expenses incurred were necessarily very heavy. Secretary, Mr. J. W. Long.

### FEVER HOSPITALS.

**London Fever,** Liverpool Road, N.—Excellently managed, providing accommodation of so good a kind that the children of many well-known citizens of position have been treated within its paying wards. It deserves well of the public, and especially of householders of all classes, and its income, £12,000 a-year, might with advantage be doubled. A great strain has been put upon the resources of this hospital lately by the prevalence of infectious diseases. Though the patients' fees cover many of the expenses, still funds are greatly needed; not only to meet present wants, but to provide extra accommodation. The domestic servants of governors and employés of subscribing firms, clubs, and hotels are treated free. Secretary, Major W. Christie; Matron, Miss F. H. Ingall.

**Ambulances.**—The London Fever Hospital provides fever ambulances for cases seeking admission. It may also be useful to state that the Metropolitan Asylums Board maintains the following ambulance stations, to which application can be made at all hours: *The Eastern,* The Grove, Homerton, E.; Superintendent, Mr. W. Robinson. *South-Eastern,* Old Kent Road; Superintendent, Mr. T. S. Plumber. *Western,* Segrave Road, Fulham; Superintendent, Mr. W. Craig. *River Ambulance Service,* South Wharf, Rotherhithe Street, S.E.; Superintendent, Mr. C. Thomson.

### NERVOUS DISEASES, EPILEPSY, AND PARALYSIS.

**Hospital for Epilepsy and Paralysis,** Portland Terrace, Regent's Park, N.W.—Great changes and improvements have been introduced of late at this institution. Mr. Howgrave Graham, the secretary, has devoted all his energies to its advancement, and has achieved a gratifying success. It is now free from debt, and £500 a-year additional income would enable its managers to aid many cases which cannot be dealt with at present for want of funds. Matron, Miss Foster.

**National Hospital for the Paralysed and Epileptic,** Queen Square, W.C.—So admirably administered in all its departments that no one interested in hospitals should fail to pay it a visit. The friends of patients afflicted with any of the dire diseases here treated, who are in a position to pay, should endeavour to secure their admission to the paying wing. Some months ago we rightly described this as a splendid special hospital (vol iv., page 307). We confidently stated out of fulness of knowledge, based upon the closest examination and inquiry, that this was an ideally perfect special hospital. For these reasons, Mr. B. Burford Rawlings, the most able Secretary, should receive many visitors and have to acknowledge numerous large gifts of money during the next few weeks. Matron, Miss L. C. East.

### THROAT AND EAR HOSPITALS.

**Central London.**—This institution in Gray's Inn Road is unendowed; over 5,000 out-patients and 300 in-patients are treated yearly. No letters of recommendation are needed, and immediate free admission is given to the poor, whilst those in a better position subscribe according to their means. There is a Samaritan fund connected with the hospital, which gives many patients the benefit of change of air, so that they may become thoroughly convalescent. Donations will be thankfully received by the Secretary, Mr. Richard Kershaw; Matron, Miss M. Danter.

**Hospital for Diseases of the Throat,** Golden Square, W.—This institution relieves 184 in-patients and 4,000 out-patients yearly. It is unendowed, but most of the patients pay according to their means. Help is needed to cover the expenses of the necessitous patients who are unable to subscribe at all, and who frequently here seek treatment when afflicted with disease. Donations in aid of the Extension Fund are also requested. Secretary, Mr. W. Thornton-Sharp, B.A.; Hon. Secretary, Mr. George C. Willoughby; Matron, Miss Phoebe Winn.

### TEMPERANCE AND CANCER.

**London Temperance,** Hampstead Road, N.W.—Patients are treated at this hospital, which is economically administered—almost a new building—without alcohol. In these temperate times one great temperance hospital should not languish for want of funds. It derived, however, only £270 per annum from invested property last year, and the whole of the balance, some £5,000 more, has to be raised annually, and, we regret to add, its income is at present less than its expenditure. This should not be, and we hope that those who believe in the non-alcoholic treatment will back their opinion by subscribing liberally at once. Hon. Secretary, Dr. Dawson Burns; Secretary, Mr. T. Mundy; Matron, Miss S. E. Orme.

**Cancer Hospital,** Brompton.—This free hospital, founded in 1851, shelters over six hundred sufferers from this most painful disease; it also benefits a large number of out-patients. With an expenditure of £29,000 the assured income only amounts to £762, so that annual subscriptions and donations are urgently needed. The Archbishop of Canterbury lately preached a sermon on behalf of this institution, and strongly recommended it to the charity of his flock. Secretary, Mr. W. H. Hughes; Matron, Miss A. Rodgers.

### HOSPITALS FOR INCURABLES.

**British Home for Incurables**, Clapham. (Office, 73, Cheapside, E.C.)—Helpless! Hopeless!! Homeless!!! These pathetic words form the motto of the British Home for Incurables, at Clapham, which national charity, in addition to providing for the nursing and comfort, but *not expensive luxury*, of the suffering inmates of the home, gives no less than £6,000 a-year in pensions to those incurably afflicted in all parts of the United Kingdom. The general funds are very low this Christmastide. In addition the sum of £15,000 is required to rebuild the home, rendered necessary by the expiration of the lease of the present premises, towards which one half has already been received or promised. Donations for either fund will be gladly received by the Treasurer, Mr. F. A. Bevan, at the Bank, 54, Lombard Street, E.C., or by R. G. Salmond, Secretary, at 73, Cheapside, E.C., where all particulars can be obtained. We are glad to hear that Mr. Salmond has been able to resume his duties with improved health. The strain cast upon him by the work in connection with the Danish Exhibition, which proved so helpful to this institution, unfortunately placed its able secretary *hors de combat*. He is so identified with its popularity and progress in the eyes of the public that it would have been disastrous had Mr. Salmond not been able to return to work, as we rejoice to know he has now done. This charity does not participate in either Hospital Saturday or Sunday Funds. Besides those who are sheltered and cared for in the home, there are three hundred incurable sufferers each receiving £20 a-year. It is scarcely necessary to lay stress on the claim these stricken brethren have on our sympathy and strength.

**Royal Hospital for Incurables**, West Hill, Putney Heath. (Office, 106, Queen Victoria Street, E.C.)—An admirable and indispensable charity of long standing, which needs, and should undoubtedly receive, much more money from the charitable. This well-known hospital is open to inspection every day but Sunday, and speaks for itself to those who will go and see it. For persons wanting a home an asylum is provided; medical attendance, nursing, and domestic comforts are supplied, and the endeavour is made to alleviate suffering and to cheer the life from which health has departed. To persons having a home, but without the means of support, a pension of £20 a-year is given; thus the family circle is unbroken, and the invalid is relieved from the pain of dependence. There are at present 213 inmates and 553 pensioners, making a total of 766. A seaside house has been opened at 55, Marina, St. Leonards-on-Sea. Subscriptions are asked specially at this season of the year, and will be thankfully received at the office, 106, Queen Victoria Street, by the Secretary, Mr. F. Andrew.

### MISCELLANEOUS SPECIAL HOSPITALS.

The following institutions have received grants from the hospital Sunday Fund during the year, are all in need of funds, and will doubtless come in for a share of the Christmas offerings. We have not space to do justice to each in detail. A fuller account, for which we are indebted to the authorities, will be given in one or two instances.

### Ophthalmic Hospitals.

Central London, 238a, Gray's Inn Road.—Secretary, Mr. William Abrahams; Matron, Miss Walsh.

Royal London, Moorfields, E.C.—This is the oldest and largest of the eye infirmaries, and grants free admission to the afflicted poor whose wants are gratuitously and liberally supplied, Assured income £1,000, expenditure £6,000. In-patients 2,049, out-patients 26,211. Secretary, Mr. Robert J. Newstead; Matron, Mrs. Peel.

Royal South London, St. George's Circus, S.E.—Secretary, Mr. Charles Comyn; Matron, Mrs. Dibsdale.

Royal Westminster, King William Street, W.C.—Secretary, Mr. T. Beattie-Campbell; Matron, Miss Rous.

Western, 153, Marylebone Road.—Secretary, Mr. G. T. Manton.

### Dental Hospitals.

Dental, Leicester Square, W.C.—Free on application; nine to eleven daily. Secretary, Mr. J. Francis Pink.

National Dental, 149, Great Portland Street.—Secretary, Mr. Arthur J. Klugh.

### Orthopædic Hospitals.

City Orthopædic, 27, Hatton Garden, E.C.—For the gratuitous surgical treatment of poor persons of every nation suffering from club-foot, contractions, and distortions of the limbs, curvatures of the spine, or other bodily deformities. Secretary, Mr. Ernest Dereuth. Matron, Miss Pollard.

National Orthopædic, 234, Great Portland Street, W.—A special appeal is made for donations towards rebuilding and enlarging this useful institution. Over 62,000 patients, children and adults, have been under treatment. The accounts are periodically audited by a firm of public accountants, appointed annually by the subscribers. £4,000 is urgently required for a new building ere the present lease falls in. Secretary, Mr. Herbert Canning; Matron, Miss S. Barnacle.

Royal, 297, Oxford Street, W.—Founded in 1838, this institution maintains 50 beds, relieving 134 in-patients, and 1,132 out-patients annually, at a cost of about £2,000. Secretary, Mr. Benjamin Maskell; Matron, Miss E. Sibley.

### Hospitals for Skin Diseases.

Hospital for Diseases of Skin, 52, Stamford Street, Blackfriars, S.E.—Secretary, Mr. Samuel Hayman; Matron, Miss Chinn.

### Fistula.

St. Mark's, City Road.—Secretary, Mr. Arthur Leared.

### Lock.

London Lock Hospital, Westbourne Green, Harrow Road.—An urgent appeal for £6,000 is issued by this hospital. There is no endowment fund, and the annual expenditure is £4,721. The west wing is used as an asylum or home, where patients who are physically cured, and show signs of moral improvement, are kept and trained in house work and helped to find situations on leaving. Secretary, Mr. A. C. P. Coote; Matron, Miss A. Lovett.

### Scrofula.

Royal Sea-bathing Infirmary, Margate. (Office, 30, Charing Cross.)—This institution is indispensable to the medical profession, as it is the only hospital of the kind in the whole country, where a certain class of distressing diseases can be treated under conditions conducive to their recovery. It contains 220 beds, and it would be well for many poor sufferers if they were doubled to-morrow. It was the special care of the late Sir Erasmus Wilson, and needs more money, which should be feely given. Secretary, Mr. J. T. Walker.

### Various.

London Homœopathic Hospital.—Contains ninety beds, supported entirely by voluntary contributions. This hospital not only benefits about 15,000 patients annually, but forms a school of medicine, and a training school for nurses. Secretary, Mr. G. A. Cross; Lady Superintendent, Miss Brew.

National Hospital for Diseases of the Heart, 32, Soho Square.—Established in Soho Square in 1857, this special hospital treats 90 in-patients and 2,000 out-patients yearly. It is unendowed, and the expenditure amounts to about £2,000 per annum. Secretary, Captain F. Handley; Matron, Mrs. Roberts.

### Lunatics and Idiots.

ST. LUKE'S HOSPITAL FOR LUNATICS, Old Street, E.C.—This asylum, situated in the very heart of London, has had 23,000 patients under its charge since its foundation, and has discharged 10,000 cured. Patients are admitted gratuitously, or on payment of a small fee according to their circumstances. The committee appeal for donations to support the institution. Secretary, Mr. Percy de Bathe, M.A.

ASYLUM FOR IDIOTS, Earlswood. (Office, 36, King William Street, E.C.)—This institution fulfils a useful purpose in quiet business-like fashion, which should commend it to the sympathies of the public. It is supported mainly by voluntary contributions, and requires additional assistance. Secretary, Mr. Jas. Downing.

### MEDICAL AND SURGICAL APPLIANCES.

**Metropolitan Hospital Sunday Fund**, Mansion House, E.C. Secretary, Mr. Henry N. Custance.—Any clergyman or minister who has a collection in his church for this fund can secure surgical appliances of every description for members of his congregation. Free surgical aid is thus granted to the poor throughout the metropolis to the extent of the funds available, but so many necessitous and distressing cases come daily under observation, that special donations, which can be sent to the Lord Mayor, are needed by the council to enable them to utilise this department of work to its fullest extent. Cheques should be crossed Bank of England.

**City of London Truss Society**, 35, Finsbury Square, E.C.—Patients to the number of over nine thousand were aided by this society last year, and they included persons of all ages from a baby a month old to adults over eighty. The work of this society is to a great measure preventive, and its timely help keeps many cases out of our hospitals. Surgical appliances are always expensive, and the poor cannot hope to obtain them without the aid of some such society as this. Secretary, Mr. John Whittington.

### PAY AND HOME HOSPITALS.

**Bolingbroke House**, Wandsworth Common, S.W.—This useful institution, where anyone living in lodgings can obtain skilled medical treatment and nursing for a small inclusive weekly payment, owes its origin to the Rev. Canon Erskine Clarke, who has borne the burden of its maintenance without complaint for nearly nine years. What that burden is may be realised by an examination of the trustees' establishment account, which shows the sum due to the trustee, Canon Clarke, at September last, to be upwards of £3,200. "Why does he go on losing his money?" we hear someone remark. "Because," answers the report, "it would betray a lack of faith in the scheme which has been upheld under many difficulties for nearly ten years to withdraw from the effort now." What is the work, then? Again the report answers: "The relief of middle-class sufferers, to whom it would be aggravation of their trial to seek admission to the general wards of a charitable hospital." Who will not feel that it would be indeed deplorable if Bolingbroke House had to be given up for lack of financial support. Its utility is being found out by the patients, who are increasing in numbers, and who have paid a larger sum (£947) this year than ever before. If, as we hope, some rich men, and possibly some captious critics, may read this week's issue of THE HOSPITAL, and arrive at this point, we would ask them is it right, is it creditable to London's wealthy citizens, and ought it further to be tolerated that Canon Clarke should still remain longer out of pocket to the tune of £3,000, because, busy man that he is, he has nobly stepped into the breach to do a necessary work for the people of London, when no one else would undertake it? We trow not. And whatever else our many hours devoted to the endeavour to set forth, fairly and fully, the needs and claims of the principal metropolitan medical charities may do, we earnestly hope that this £3,000 will be sent to Canon Clarke during the next few weeks. Resident Medical Officer and Secretary, Dr. Cecil R. C. Lyster.

**Hampstead Home Hospital and Nursing Institute**, Parliament Hill Road, N.W.—This is a useful institution, which has done a great deal of good. It is excellently managed, well found, comfortable, and homely. It provides medical and surgical treatment on payment of from seven shillings to five guineas weekly, and also sends out trained lady nurses to private families. A payment of seven shillings a-week may enable patients to retain their self-respect, but it does not defray the cost of their maintenance, so those who sympathise with the hard lot of teachers, clerks, shop assistants, and others similarly situated, may like to send a contribution to Mr. R. A. Owthwaite, the Secretary.

### DISPENSARIES.

**Provident and General Dispensaries.**—There are not less than fifty of these institutions scattered all over the metropolis, and depending mainly for their support on the free offerings of the charitable who live in the district, to the poorer inhabitants of which they render such material service. We have not space to name all these institutions, and it would be unfair to name some where all are so admirably administered. We call special attention to them, however, because they should recommend themselves to the especial care of the better classes, who are indebted to them for the great services they render, in the time of sickness and suffering, to their poorer neighbours. If any reader should be unaware of the address of the dispensary in his district, let him send to the Mansion House for a copy of the last report of the Hospital Sunday Fund, where he will find a full list of the institutions to which we refer.

### DISTRICT AND OTHER NURSING INSTITUTIONS.

In an article in another column, entitled "Hospitals at Home," we remark: "It must be remembered that new departures, which involve the expenditure of public money, or the interference, in however small a degree, with vested interests, must always be asked to show cause." Now, to our knowledge, there are few more useful or necessary organisations in modern times than the nursing institution, which may be said to bring the care, the skill, and the comfort of the hospital ward to the home of the poor in the day of sickness. We would gladly have called attention to the claims on public sympathy possessed by the many useful nursing institutions to be met with in and around London. No such appeals have, however, reached us, and, whereas we hope to be able to give some account of their aims and objects in THE HOSPITAL ANNUAL, we would venture to hint to the managers that in future years it might be well for them to show cause at this season why some of the Christmas offerings should be poured into their coffers.

Since the above was written, Miss Wilson, the honorary secretary of the Workhouse Nursing Association, writes from 45, Colville Gardens, W., to say how urgently she needs £1,000 to enable her Association to do properly the very useful work which it undertakes, viz., the introduction of skilled nursing into Poor-law infirmaries throughout the country. This should be subscribed at once, when it is realised that some guardians consider £12 per annum high wages for a pauper nurse (?) Why should not sick paupers be properly tended? Who will not subscribe to secure this?

### HOSPITAL SATURDAY FUND.

The objects of this fund are the collection of subscriptions from clerks, artisans, mechanics, shop, assistants, and others, who may not be reached by the Sunday Fund, chiefly by means of collections in the various workshops or by boxes for distribution amongst the hospitals. We desire to remind our readers of these objects at this season because there may be some of them who, though unable to give much money, might be willing to give some time to the cause of the medical charities. If this is the case, we would suggest their communicating with Mr. Robert Frewer, Secretary to the fund, 5, Mitre Court, Fleet Street, E.C. Mr. Frewer has displayed great ability and devotion in his work, and his influence has had a marked effect upon the administration of the fund, and consequently upon the position it now occupies in public esteem. He would, we are certain, welcome the co-operation of any energetic workers who may desire to help him in his arduous undertaking.

### A FEW OTHER CHARITIES.

The following information has been sent us about the institutions mentioned, which we are glad to publish :—

**Field Lane Refuges and Ragged Schools,** Vine Street, Clerkenwell, E.C.—The committee of this charity appeal for special funds to defray the expenses of dinners given on Christmas Day to 700 of the destitute, and 600 poor families. Secretary, Mr. Peregrine Platt.

**Indigent Blind Visiting Society,** 27, Red Lion Square, W.C.—Dr. Armitage appeals for funds to cover the expense of the Christmas dinners and gifts of coals which are annually presented to the blind poor at this season. No expense is incurred in the distribution of gifts received, as voluntary helpers undertake the work of visiting and relieving those in distress. Over 900 were benefited last year. Secretary, Mr. W. C. Lester.

**London Orphan Asylum.**—The managers of this institution appeal for aid at this season to meet the year's expenses, which, owing to enlarged admissions, are very heavy. The following are particulars :—97 orphans have been received this year, and 500 are now in the asylum; 5,100 orphans have been maintained, clothed, and educated, each for an average period of six years, since the foundation of the charity. The institution depends almost entirely upon voluntary contributions. Donations will be very gratefully received by Mr. Arthur R. Capel, Treasurer, and Mr. James Rogers, Secretary, at the offices, 21, Great St. Helen's, E.C.

**Metropolitan Convalescent Institution,** 32, Sackville Street, W.—The homes of this institution are at Walton-on-Thames, Kingston Hill, and Bexhill-on-Sea. Between 4,500 and 5,000 patients, mostly of the poor class, are admitted yearly, entirely free, on the recommendation of subscribers. The institution has no endowment, and, as the cost of maintaining so great a number of patients is necessarily large, the committee are compelled to appeal to the benevolent public. Secretary, Mr. Chas. Holmes.

**Metropolitan Provident Medical Association,** 5, Lambs Conduit Street, W.C.—Provides medical treatment and medicines for the working classes, fosters habits of providence and independence, and keeps back many unsuitable patients who would otherwise crowd our hospital wards. The association is growing rapidly, and has already 13 centres and 26,000 members. To meet the extension of the movement funds are urgently needed. Secretary, Mr. W. G. Bunn.

**Mission to Deep Sea Fishermen,** Bridge House, Blackfriars, E.C.—The two hospital ships, the *Victoria* and *Albert*, belonging to this mission, are useless for want of funds. It is said that the ships are to be taken round to the Isle of Wight for the Queen to inspect, ere they first sail with the deep-sea fleet. Each boat will carry an experienced surgeon and all necessaries, for the treatment of either medical or surgical cases. There are the boats, there are the medical men, but with a horror of debt the mission refuses to send them forth till they have money in hand to cover expenses. Founder and Director, Mr. E. J. Mather.

**Royal Albert Orphan Asylum,** 62, King William Street, E.C.—Two hundred destitute orphans and fatherless children are fed, clothed, and educated at this excellent asylum. The girls are trained as servants, the boys are taught trades; and when old enough, situations are sought for them. The chief point about this institution is the non-canvassing system of voting, which saves the usual heavy outlay in stamps and stationery. The asylum is entirely dependent upon voluntary contributions, and funds are earnestly solicited. Secretary, Mr. Richard Witherby.

**Thames Church Mission,** 31, New Bridge Street, E.C.—An appeal is issued by this excellent mission for funds to send the new steam launch *Edward Auriol* to her work. The preaching of the Gospel to the floating population of the Thames is hindered for want of means. Secretary, Rev. H. Bloomer.

